# Enhancing SNV identification in whole-genome sequencing data through the incorporation of known genetic variants into the minimap2 index

**DOI:** 10.1186/s12859-024-05862-y

**Published:** 2024-07-13

**Authors:** Egor Guguchkin, Artem Kasianov, Maksim Belenikin, Gaukhar Zobkova, Ekaterina Kosova, Vsevolod Makeev, Evgeny Karpulevich

**Affiliations:** 1 Ivannikov Institute for System Programming, Moscow, Russia; 2 Institute for Information Transmission Problems, Moscow, Russia; 3 Evogen LLC, Moscow, Russia; 4 Vavilov Institute of General Genetics, Moscow, Russia; 5 Institute of Biochemistry and Genetics of Ufa Scientific Centre, Ufa, Russia; 6 Present Address: Cancer Research UK National Biomarker Centre, University of Manchester, Manchester, Manchester, M20 4BX UK

**Keywords:** Minimap2, Minimap2 index modification, Read alignment, Human pangenome, HPRC, NGS

## Abstract

**Motivation:**

Alignment of reads to a reference genome sequence is one of the key steps in the analysis of human whole-genome sequencing data obtained through Next-generation sequencing (NGS) technologies. The quality of the subsequent steps of the analysis, such as the results of clinical interpretation of genetic variants or the results of a genome-wide association study, depends on the correct identification of the position of the read as a result of its alignment. The amount of human NGS whole-genome sequencing data is constantly growing. There are a number of human genome sequencing projects worldwide that have resulted in the creation of large-scale databases of genetic variants of sequenced human genomes. Such information about known genetic variants can be used to improve the quality of alignment at the read alignment stage when analysing sequencing data obtained for a new individual, for example, by creating a genomic graph. While existing methods for aligning reads to a linear reference genome have high alignment speed, methods for aligning reads to a genomic graph have greater accuracy in variable regions of the genome. The development of a read alignment method that takes into account known genetic variants in the linear reference sequence index allows combining the advantages of both sets of methods.

**Results:**

In this paper, we present the minimap2_index_modifier tool, which enables the construction of a modified index of a reference genome using known single nucleotide variants and insertions/deletions (indels) specific to a given human population. The use of the modified minimap2 index improves variant calling quality without modifying the bioinformatics pipeline and without significant additional computational overhead. Using the PrecisionFDA Truth Challenge V2 benchmark data (for HG002 short-read data aligned to the GRCh38 linear reference (GCA_000001405.15) with parameters k = 27 and w = 14) it was demonstrated that the number of false negative genetic variants decreased by more than 9500, and the number of false positives decreased by more than 7000 when modifying the index with genetic variants from the Human Pangenome Reference Consortium.

**Supplementary Information:**

The online version contains supplementary material available at 10.1186/s12859-024-05862-y.

## Introduction

Sequencing technologies are becoming less expensive, and there is an inevitable transition from sequencing individual genomes to mass sequencing of individual populations. The most famous project is a study of the genomes of UK residents. At the moment, 500 thousand complete genomes of UK residents have been sequenced and clinical metadata have been collected for these people, which not only allows the problems of individual genomics to be solved, but also makes it possible to conduct genome-wide association studies to identify characteristic variants associated with a particular phenotype that are not related to individuals, but belong to a group of people or a specific population [1]. The massive use of sequencing technologies makes it urgent to create high-quality methods for analysing the output data of a sequencer, as well as the use of information about already known genetic variants of a particular population in the creation of such methods.

Human whole-genome sequencing is currently performed predominantly using Next-Generation Sequencing (NGS) technology [2]. When analysing NGS whole-genome sequencing data, one of the key steps is the alignment of reads to a reference genome sequence.

**Fig. 1:**
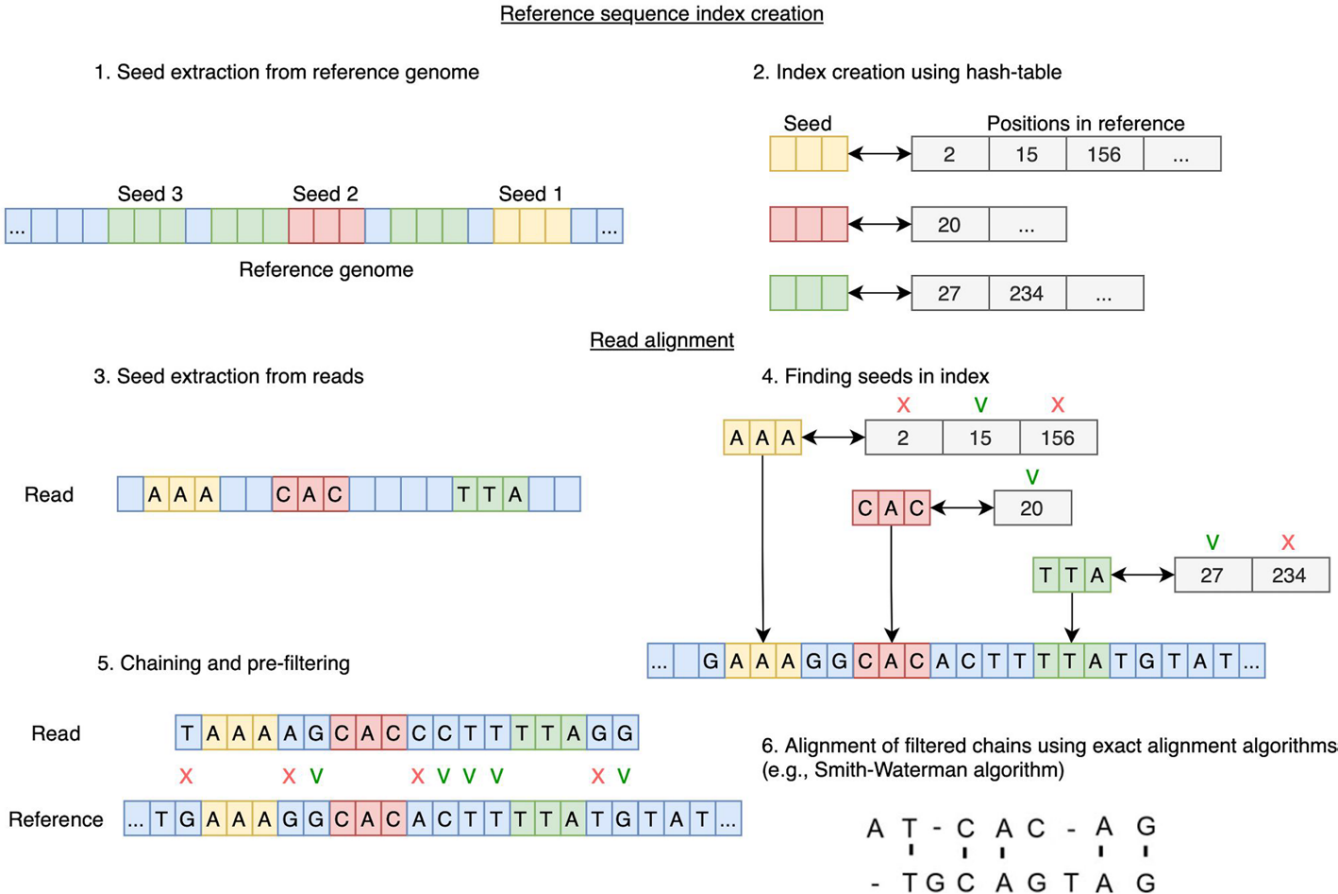
Read alignment with seed-and-extend approach. Initially, a reference sequence index is created. The first step is to compute all seeds from the reference sequence, the second step is to store all the seeds in a convenient data structure (e.g. a hash table). For read alignment, seeds are also computed from its sequence (step 3), which are then mapped to the seeds from the index (step 4). Sequential seed chains are then calculated (step 5) using the matched reference and read seeds. In the final step, an exact alignment is performed for the chain with the highest score

The most commonly used alignment approach is the two-step seed-and-extend [2–4] approach (Fig. 1). It is based on partitioning reads and the reference genome sequence into relatively small subsequences of nucleotides (seeds) [5], finding exact matches (anchors) between reads and reference sequence seeds, identifying sets of collinear anchors as chains, and then aligning them using alignment algorithms such as the Smith-Waterman [6] or Needleman-Wunsch [7] algorithms. A number of tools use a Burrows-Wheeler Transform (BWT) suffix array to find anchors [8], using an FM-index [9] to speed up substring searches in the suffix array. The popular minimap2 alignment tool uses a seed-and-extend alignment approach similar to the one described in the original paper, called seed-chain-align, and a different index structure known as a sampled suffix array (SSA) [10]. The SSA is a sparse representation of a suffix array, a data structure used to efficiently search for substrings in strings. SSA is similar in structure to a hash table.

The presence of genetic variants in a short-read sequence generally reduces the mapping quality (MAPQ) [11] score of an aligned read or reduces the likelihood of determining the position of the read correctly. In addition, there are complex regions of the genome, such as repeats, for which the alignment of short reads remains an open problem [12]. One possible approach to aligning reads to complex regions of the genome would be to create a genomic graph [13], which is constructed by taking into account the known genetic variants. At present, the construction and further application of the genomic graph of the human genome and its index is a complex computational task, with high memory requirements for the storage and computation of the genomic graph [14].

Another approach for solving the problem of aligning reads with indels is to use sets of related k-mers. These k-mers are spaced at some distance between each other, forming gaps in the sequence. The gaps allow reads containing indels to be aligned with a higher probability [14–16].

In this paper, we developed a new way to construct a linear reference sequence index that takes into account known genetic variants using the features of the internal representation of the reference sequence index of the minimap2 tool. The reference sequence remains linear, and the index is augmented with minimizers containing the known genetic variants, thus eliminating the need for significant additional computations when aligning short reads.

## Materials and methods

**Fig. 2:**
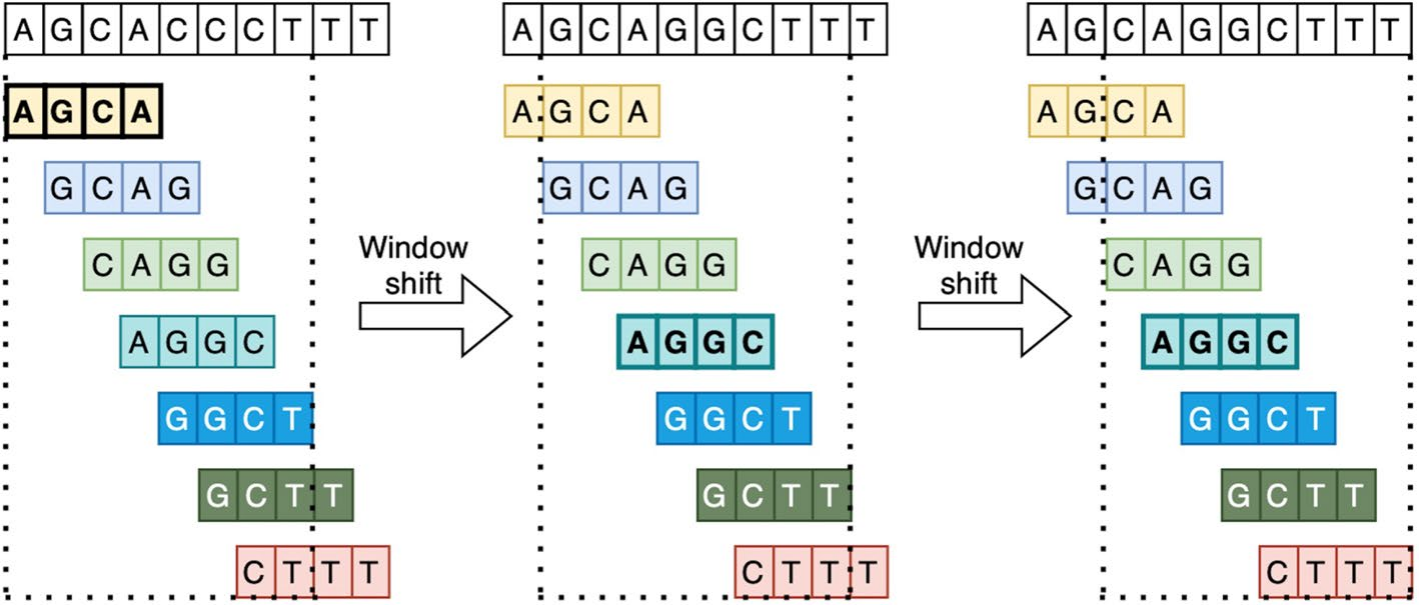
Computation of minimizers with length 4 in windows of length 5. Assuming that the hash function results from the sequence are arranged in the lexicographic order of the original sequences, the sequence “AGCA” will be the minimizer in the first window, and “AGGC” will be the minimizer in the second and third windows

One of the key features of the minimap2 tool is the use of minimizers [15]. A minimizer is a short substring (or, otherwise, k-measure) that is located within a given sequence. The term “minimizer” comes from the idea of minimizing the number of substrings that need to be compared during the alignment process. A minimizer is calculated by applying a given hash function to each k-mer in a given sequence window to create a unique integer value. The integer values are then sorted and the k-measure with the smallest value is selected as the minimizer (Fig. 2). Using minimizers results in a faster alignment process [16].

When indexing a linear reference sequence, a hash table is created where keys are the results of applying the hash function to the minimizer sequence and values are the list of positions in the genome reference sequence.

**Fig. 3:**
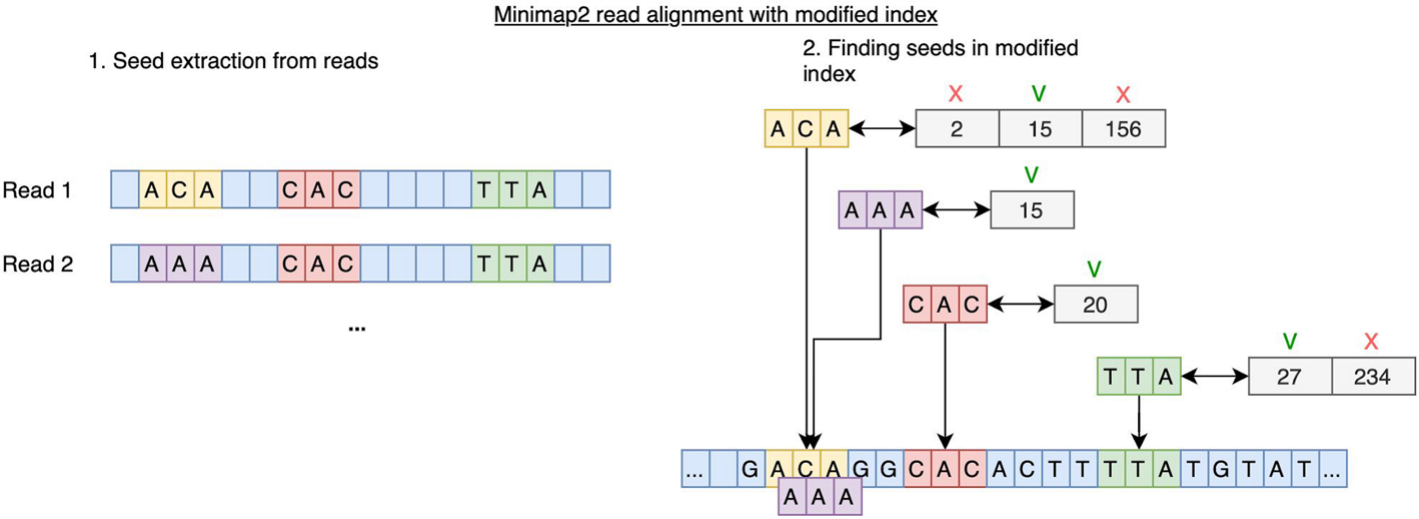
Minimap2 read alignment with modified index. In the first step, seeds are extracted from the read sequences. For this example, read 1 contains the seeds “ACA”, “CAC”, “TTA”; read 2 contains SNV in the third position (C– > A), and so instead of the seed “ACA” it has the seed “AAA”. In the second step, the seeds from the read sequences are matched to positions from the hash table created from the reference genome. The SNV from the second read is assumed to be incorporated in the modified index, so the hash table contains the seed “AAA” with position 15

The possibility of modifying the minimap2 tool index is provided by the fact that the hash table does not impose any restrictions on the number of minimizers at a given position of the linear reference sequence, and adding information about genetic variants does not affect the subsequent alignment algorithm. In other words, the linear reference sequence index allows the addition of branches induced by the addition of genetic variants, similar to a genomic graph (Fig. 3).

The minimap2_index_modifier tool reads known genetic variants (single nucleotide variants and indels) from the Variant Call Format (VCF) file and modifies the index to take these genetic variants into account. Using a single SNV as an example. For each genetic variant, a window of fixed length 2 * (k + w)−1 is defined (where k and w are the length of the k-mer and the length of the window specified during the index computation, on which the k-mer with the minimum hash function result is computed). For this window, a subsequence of the original reference sequence is taken and the central element of this window is changed according to the genetic variant. The range of the window is determined by the algorithm for finding minimizers: the sliding window identifies all k-mers with the minimum calculated hash function value within the window. As soon as such k-mers go beyond the window boundaries, they become minimizers, and the algorithm searches for new k-mers in the shifted window. By setting the window length to (2 * (k + w)−1), we ensure the correct computation of minimizers that contain genetic variants. The new minimizers, which contain the genetic variant, are then added to a common hash table. The tool's standard behavior is to add minimizers for all given SNVs.

The process of adding indels is more complex than that of adding SNVs. The main difference is that for deletions, the minimizer position must be shifted by the difference of the ‘deleted’ subsequence.

During the implementation of indel addition, interesting observations were made. A BAM/SAM file containing aligned reads includes a Compact Ideosyncratic Gapped Alignment Report (CIGAR) [17] field for each read. For example, read alignment to the modified index of reads with indels in the middle of the sequences showed only matches (M) in the CIGAR field, indicating that the read is fully aligned without any indels. These observations demonstrate that utilizing the index modified by our tool enables the consideration of short indels.

Examples of databases containing known genetic variants include dbSNP [18], gnomAD [19], the 1000 Genomes Project [20] and the Human Pangenome Reference Consortium. All of these databases can be used to modify the linear reference sequence index. The 1000 Genomes Project dataset contains phased genotypes, which allows us to consider only those genetic variants that occur together. For this purpose, our tool can iteratively consider all windows around genetic variants and compute new minimizers from the resulting haplotypes. If a genetic variant is unphased, all linear combinations of nearby genetic variants are computed. If there are too many such genetic variants (more than ten in a given window), such windows are ignored toтaм avoid generating too many minimizers. Windows with indels overlapping other SNVs are also ignored.

## Results

To evaluate the efficiency of the reference sequence index modification, a series of experiments were performed. The modified index contained both SNVs and indels in all experiments except for the one involving population-specific genetic variants. Reference sequences of different versions, namely GRCh38 and GRCh37, were used. Various genetic variant databases were also used for index modification. All experiments were conducted using a computer with two 32-core processors (128 threads) operating at a frequency of 2.9 GHz.

### Incorporating genetic variants into the GRCh38 reference

To evaluate the effect of modifying the linear reference sequence index of the GRCh38 *(GCA_0000000001405.15)* version, two versions of the modified indexes were created. In the first version frequently occurring genetic variants with minor allele frequency greater than 0.05 (the value was chosen based on the results of the experiment, more details in the Supplementary Table 1) from the 1000 Genomes Project (Phase 3) were added to the standard index, taking into account possible haplotypes. For the second version, publicly available assembly for 44 samples (excluding HG002) from the HPRC was used. A series of whole-genome sequencing experiments were performed on paired-end reads with a length of 150 nucleotides for HG002 with 35X coverage from the PrecisionFDA Truth Challenge V2 [21]. The reads were preprocessed using the Cutadapt tool [22].

The index was created using the recommended values of k = 21 and w = 11 for aligning short reads. In general, larger values of k lead to a higher probability that reads not containing genetic variants will be aligned, but also increase the probability that reads containing genetic variants will not be aligned [23]. However, our modification of the index should mitigate the negative effect to some extent. Smaller values of k lead to an increase in the algorithmic complexity of the alignment procedure due to the increase in the total number of k-mers. In addition, when k is set to a low value, the number of unique minimizers decreases. Two boundary cases are k = 1 (4 letters) and k = 32 (index out of bounds of the array in the minimap2 code). To demonstrate that modifying the indexing of the minimap2 tool has a positive effect across different values of k and w, not only limited to non-degenerate values, additional experiments were conducted. These experiments included pairs of k and w values obtained by proportionally shifting the original values of k and w by (+ 6, + 3) and (− 6, − 3), resulting in pairs of (15, 8) and (27, 14) for k and w, respectively.

The stumbling block in parameter selection during alignment is the mapping parameter f (specified by two integers n and m) which is used to filter out the most frequent minimizers that occur more than n and m times. Such minimizers are typically derived from sequences of repeated elements (if the length of such a sequence exceeds k + w−1, then several equal minimizers will be calculated) and are ignored at the seeding stage for optimisation purposes (the second integer is used in the second round of seeding). Even slight changes in the parameter f can lead to changes in the final results [24]. However, it is unclear what values of f should be used for other sets of k and w values. Assuming that such minimizers are derived from the same regions of the reference genome, in this paper we choose the value of f such that the ratio of ignored minimizers to all other minimizers remains the same.

When conducting experiments with a modified reference genome index, the parameter f must also be changed, because adding new minimizers may change the overall ratio of minimizers to each other. To minimize the possible effect of discarding frequently occurring minimizers, for iterations of the modified index experiment, the standard values were increased so that approximately the same number of minimizers was ignored in the seeding step.

**Fig. 4:**
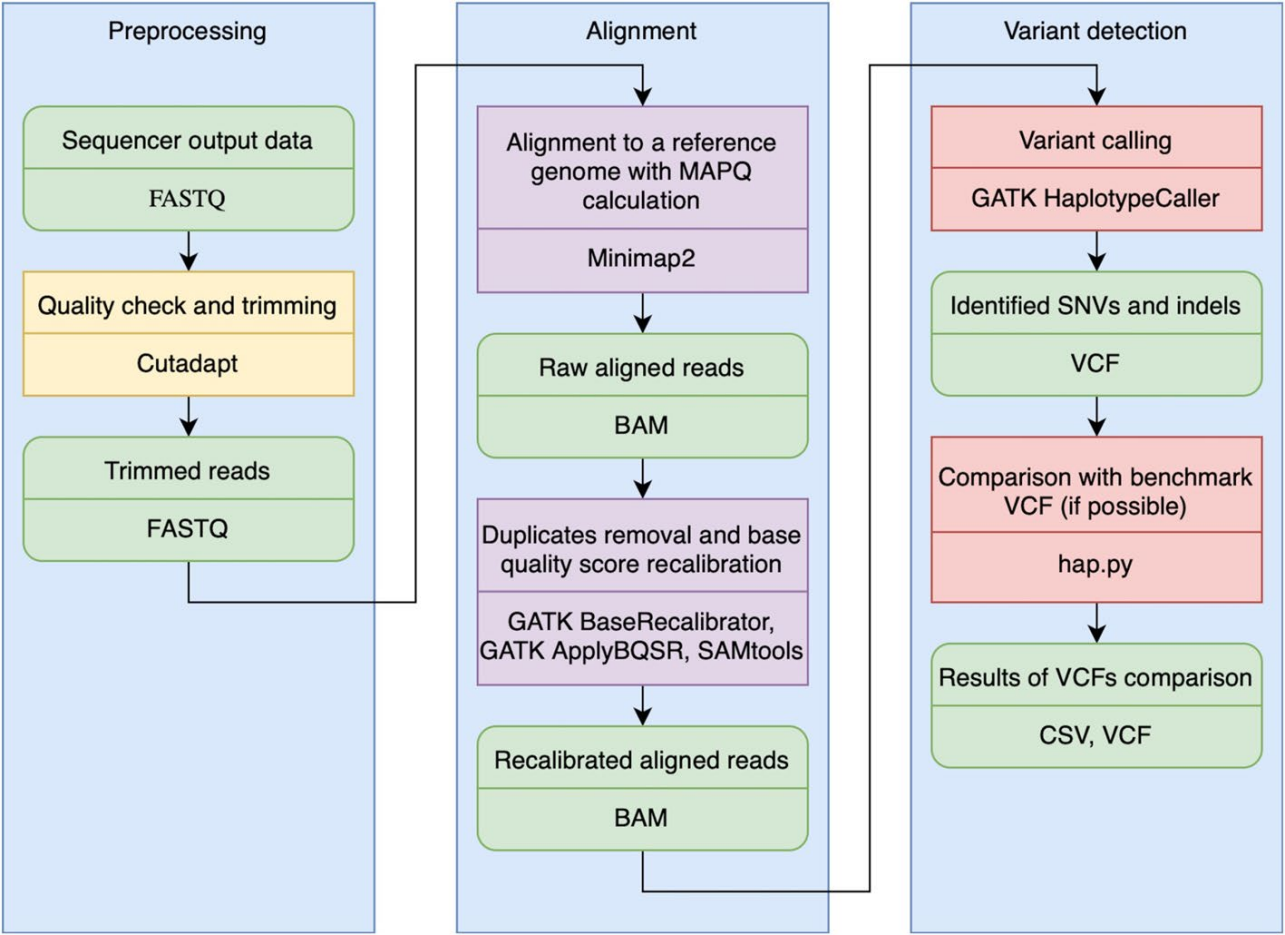
Scheme of the whole-genome sequencing pipeline

The Broad Institute’s WARP Whole Genome Germline Single Sample Pipeline [25] was used as the basis for the whole-genome sequencing pipeline. The initial stage of the pipeline was modified to obtain FASTQ-formatted data, which were subsequently aligned using the minimap2 tool. In addition to minimap2, different aligners like Bowtie2, BWA-MEM, BWA-MEM 2 were also added. The aligned reads from the Binary Alignment Map (BAM) file were further processed using the SAMtools tool [17]. Potential sequencing errors were corrected using the GATK BaseRecalibrator and GATK ApplyBQSR tools. Variants were called using the GATK HaplotypeCaller tool. The VCF files obtained from the pipeline were compared to the reference VCF files using the hap.py tool [26] (Fig. 4).

**Fig. 5:**
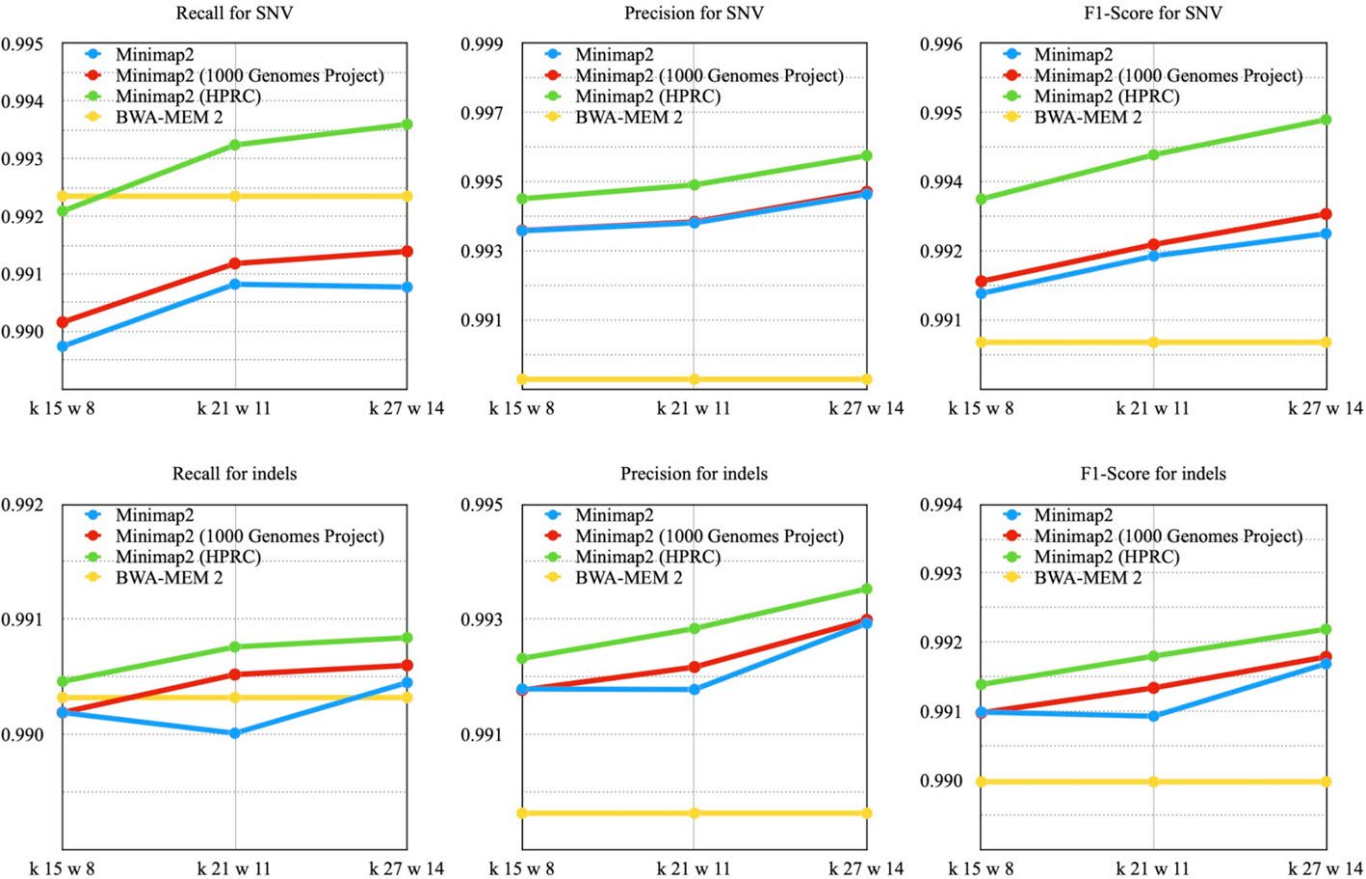
The VCF files were compared to the reference files on ‘confident regions’ using HG002 and reference data from the PrecisionFDA Truth Challenge V2. The GRCh38 linear reference sequence (GCA_000001405.15) was used

**Fig. 6:**
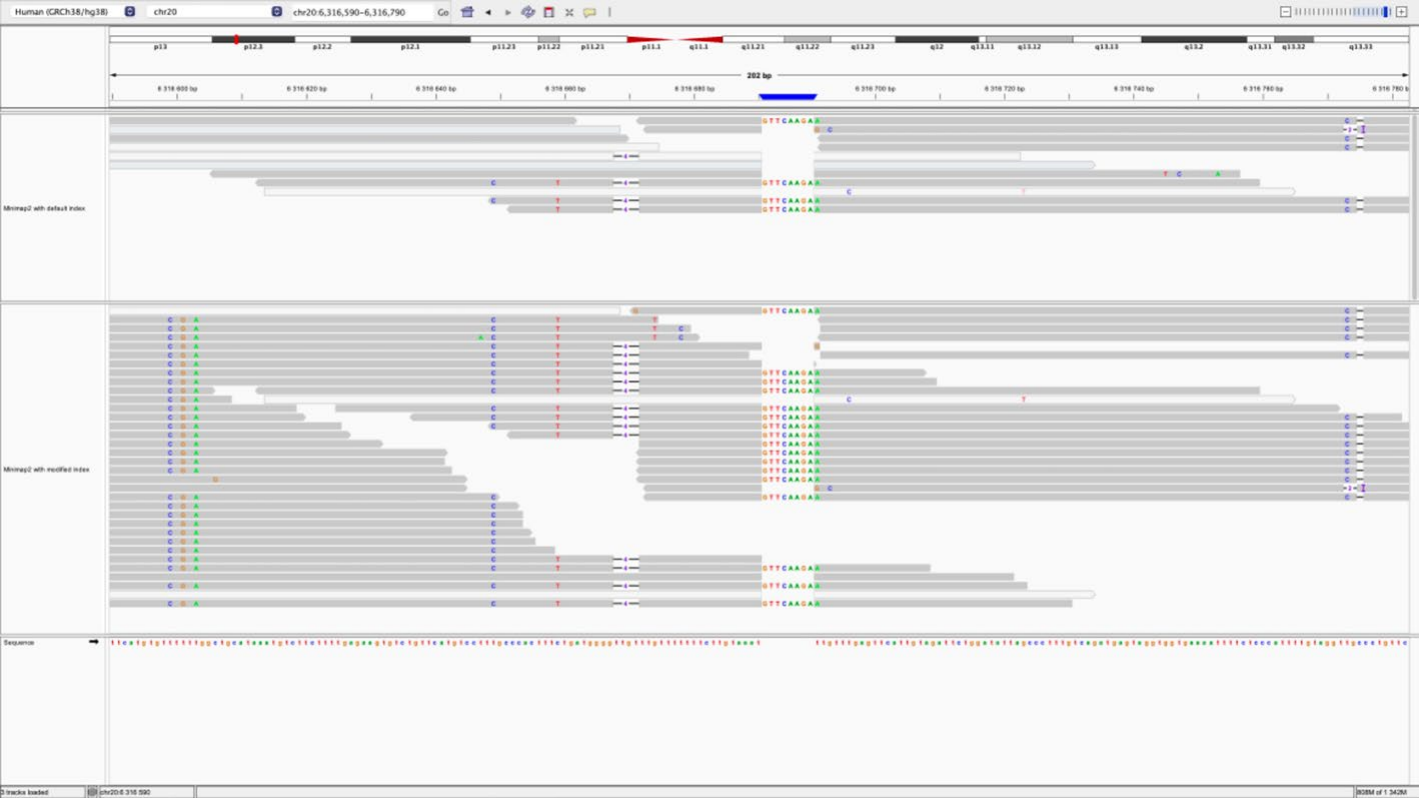
The genetic variants found using the modified index. There are 6 SNVs (chr20: 6,315,599, 6,315,601, 6,316,603, 6,316,649, 6,316,659, 6,316,773), 1 insertion (chr20: 6,316,690) and 2 deletions (chr20: 6,316,667, 6,316,774)

For all runs, the use of the modified index resulted in a reduced number of multi-mapped reads. (Supplementary Table 2). While the index modified with data from the 1000 Genomes Project showed no significant improvement, the index modified with data from the HPRC showed the best results, outperforming BWA-MEM2. As expected, larger values of k lead to larger increases in metrics and vice versa. The lower F1 score for k = 15, w = 8 may be due to the fact that long indels (with length > 15) are not incorporated into the modified index in this case, or to the poor choice of the genetic variant database. (Fig. 5, Supplementary Table 3). Figure 6 and Supplementary Figs. 1–2 denote an example of genetic variants found using the modified index.

### Incorporating genetic variants into the GRCh37 reference

Similar to GRCh38, the GRCh37 (hs37d5) linear reference sequence index was modified. The coordinates of genetic variants from HPRC’s VCF file were preprocessed using the Picard LiftoverVcf tool. Thus, some of the genetic variants were lost during conversion.

Iterations of the whole-genome sequencing pipeline were performed on PrecisionFDA Truth Challenge data. Short 150-nucleotide paired-end reads were used for HG002 at 50 × coverage, along with v4.2.1 reference BED (Browser Extensible Data) and VCF files.

Experiments performed using the GRCh37 human reference genome showed that the use of the modified index leads to an increase in all metrics for all performed runs.

**Fig. 7:**
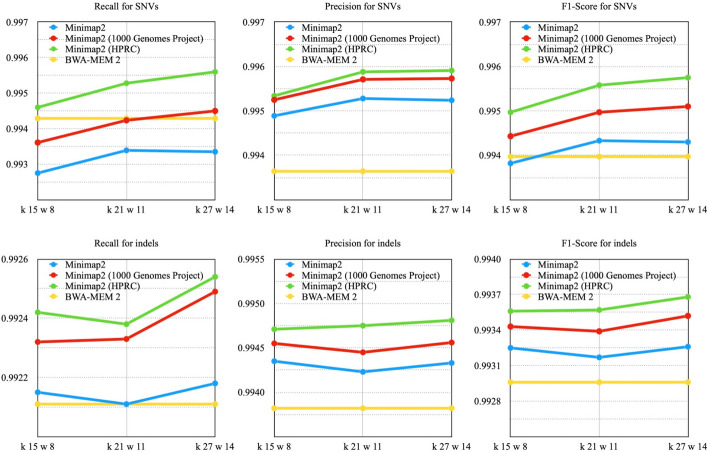
The VCF files were compared to the reference data from the PrecisionFDA Truth Challenge using HG002 as the reference. The linear reference sequence GRCh37 (hs37d5) was used

The index modified by genetic variants from HPRC outperforms the BWA-MEM2 results, for all k, w sets used. And the index modified with genetic variants from the 1000 Genomes Project showed better results than the one used for GRCh38, which may be due to both the number of genetic variants added and the database itself. (Fig. 7, Supplementary Table 4).

### Incorporating genetic variants into the GRCh37 reference for synthetic data

For this experiment, short paired-end reads of 150 nucleotides were additionally synthesized based on a 25X-coverage VCF file of the HG002 sample. The ART toolkit [27] was used for synthesis. Reads were not generated for the whole genome but were generated separately for chromosomes 1 and 6. Chromosome 1 was selected because it is the longest chromosome in the genome and chromosome 6 was chosen because it contains the major histocompatibility complex (MHC), a highly variable region. Reference BED and VCF files v4.2.1 were used for validation. In contrast to the other experiments, the standard parameters of the minimap2 tool were used to generate the reference sequence indexes.

**Fig. 8:**
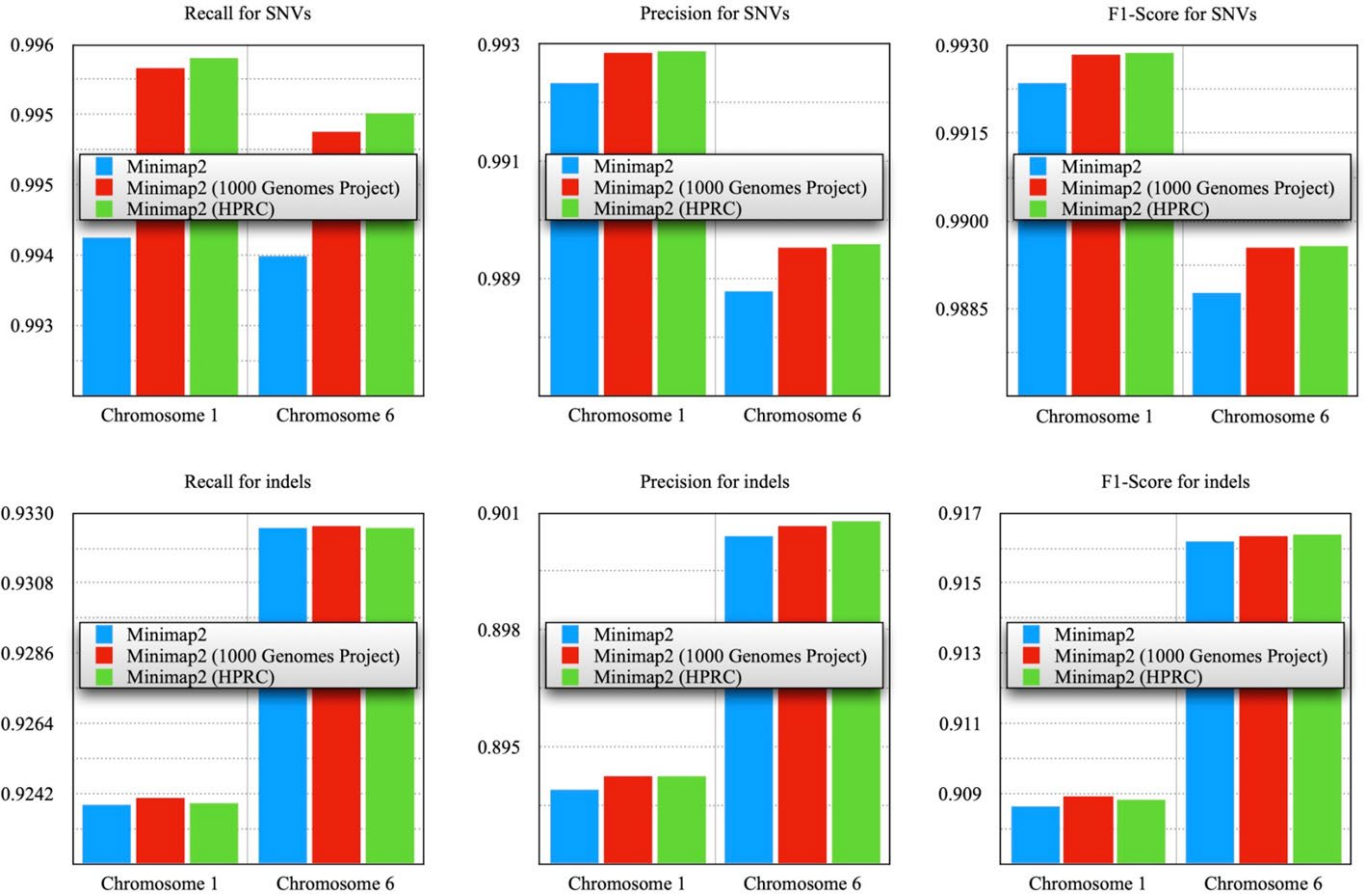
Comparison of the obtained VCF files with the reference HG002 files, synthesized paired-end reads for chromosomes 1 and 6 generated from the reference VCF file of the HG002 sample were used. The linear reference sequence GRCh37 (hs37d5) was used

As in previous experiments with real data, modifying the reference sequence index on synthetic data also resulted in higher final quality metrics (Fig. 8, Supplementary Table 5). However, experiments with synthetic data for other samples may cause difficulties as other publicly available samples may already be included in genetic variant databases, and adding variants associated with these samples will disrupt the entire validity of further experiments.

### Evaluating the effect of adding population-specific genetic variants

Previous experiments added all genetic variants with an MAF > 0.05 from the 1000 Genomes Project dataset to the reference sequence index. The dataset comprises 26 different populations, and some genetic variants are only characteristic of certain populations. In this experiment, we compared the effect of adding population-specific genetic variants on the final quality of the whole-genome sequencing pipeline. To achieve this goal, we generated several modified reference sequence indices by adding genetic variants of a specific population with AF > 0.05 and not found in other specified populations with AF > 0.05 from the gnomAD v3.1.2 dataset: Ashkenazi Jewish, Mixed American, and East Asian. As a result, we obtained three modified indices, each with non-overlapping genetic variants. The intermediate VCF files contained 158,018 single nucleotide variants (SNVs) for the Admixed American population, 311,393 for the Ashkenazi Jewish population, and 487,442 for the East Asian population. In this experiment, as in the first experiment, short paired-end reads of 150 nucleotides were used for HG002 with 35X coverage from the PrecisionFDA Truth Challenge V2, and alignment was performed to the GRCh38 reference sequence (GCA_00000101405.15).

**Fig. 9:**
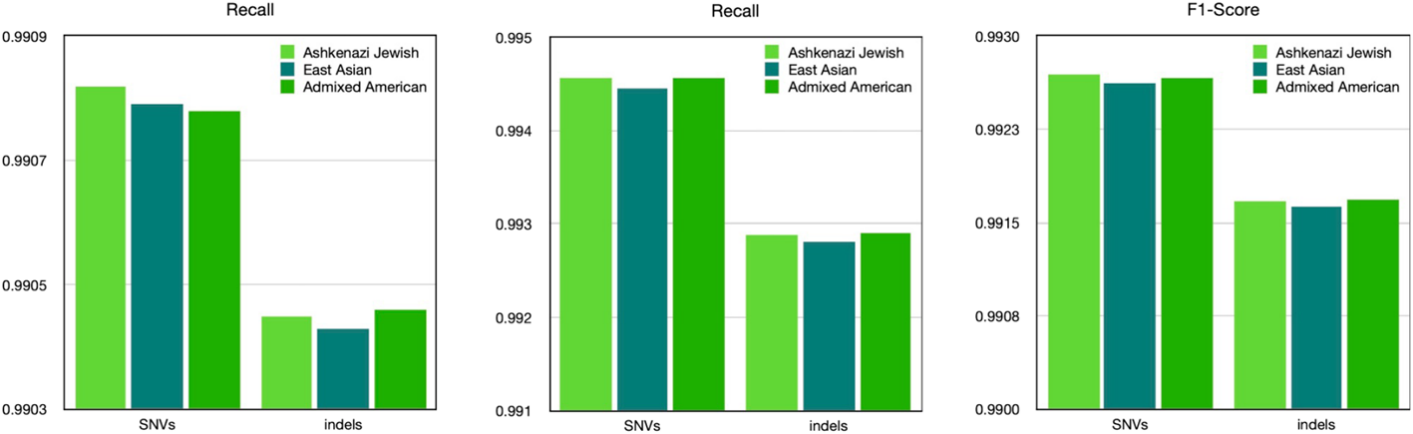
The VCF files were compared to the reference on 'confident regions' using HG002 and reference data from PrecisionFDA Truth Challenge V2. The GRCh38 linear reference sequence (GCA_000001405.15) was used. The index was modified by adding population-specific mutations from gnomAD v3.1.2

According to the experimental findings, the index incorporating mutations from the Ashkenazi Jewish population demonstrated the highest values for Recall, Precision, and F1-Score metrics concerning SNPs (Fig. 9, Supplementary Table 6). However, the results for indels were less unequivocal, which could be attributed to the exclusive use of SNPs in the modification process.

## Conclusion

The experimental results demonstrated that the use of the minimap2_index_modifier index modification tool can effectively reduce the number of undetected genetic variants. The modified index can be used in pipelines for whole-genome sequencing data analysis via the minimap2 alignment tool to improve SNV calling quality. These modifications can also be applied in the development of new read alignment methods. At the same time, users of this tool have flexibility to select the sets of genetic variants to be used for index modification, depending on the specific task at hand. This work shows that using a reference genome index modified with HPRC genetic variants provides the greatest benefit most likely due to the high quality of HPRC genetic variants at the structural and base pair levels.

Currently, the minimap2_index_modifier supports the addition of SNVs and indels that are shorter than the specified k-mer length. This is because calculating minimizers for longer indels can cause a significant shift in their positions relative to the reference genome, resulting in incorrect alignment. One potential solution for incorporating longer indels is to convert them into a separate vector whose position corresponds to the position of the initial [28, 29] symbol. However, implementing this solution would require a total revision of the existing alignment algorithm and is likely to have a negative impact on the final performance.

It may be worth changing the approach to selecting genetic variants; for example, adding genetic variants next to repetitive regions may lead to incorrect alignment of reads because the added genetic variant has caused the repetitive region to expand. Such situations were not covered in this paper.

### Supplementary Information

**Additional file 1**. Supplementary tables, figures, methods and algorithms.

## Data Availability

The source code of the minimap2_index_modifier tool is available at: https://github.com/ispras/minimap2_index_modifier. The simulation data are available at: https://nextcloud.ispras.ru/index.php/s/z3HyaZjGf8aJLYS. Minimap2 generated indexes are available at: https://nextcloud.ispras.ru/index.php/s/wcb9PpZyr8Gb5CC
