# Supplementary material for: Enhancing SNV identification in whole-genome sequencing data through the incorporation of known genetic variants into the minimap2 index
Source: BMC Bioinformatics. 2024 Jul 13;25:238. doi: 10.1186/s12859-024-05862-y (PMC11246581; doi:10.1186/s12859-024-05862-y)
Supplement: Supplementary file 1 — Additional file 1. Supplementary tables, figures, methods and algorithms. [file 12859_2024_5862_MOESM1_ESM.pdf]

Supplementary Material

1. Supplementary Results

Supplementary Table 1. Results of the allele frequency selection experiment for genetic variants from the 1000 Genomes Project.

Tool	Minimap2 with modified index (VCF from 1000 Genomes Project)				
Allele frequency	0.5	0.2	0.05	0.02	0.005
SNVs in index	1932068	4368844	7609482	10254402	16695721
Indels in index	338296	757897	1193661	1481299	2080783
k-mer and window	k 21, w 11				
Minimizers in index	491422780	499880055	511183729	520391349	542783669
Parameter f	1014, 5075	1033, 5148	1055, 5223	1073, 5286	1116, 5458
**SNVs**					
TP	3331654	3332986	3333360	3333388	3333296
FN	21032	19700	19326	19298	19390
FP	14945	14485	14379	14427	14580
Recall	0.99373	0.99412	0.99424	0.99424	0.99422
Precision	0.99553	0.99567	0.99570	0.99569	0.99565
F1-Score	0.99463	0.99490	0.99497	0.99497	0.99493
**Indels**					
TP	518314	518365	518381	518383	518388
FN	4076	4025	4009	4007	4002
FP	2936	2905	2896	2886	2900
Recall	0.99220	0.99230	0.99233	0.99233	0.99234
Precision	0.99437	0.99443	0.99445	0.99447	0.99444
F1-Score	0.99328	0.99336	0.99339	0.99340	0.99339

Supplementary Table 2. The results derived from the minimap2 tool encompassed the quantification of multi-mapped reads and aligned reads based on MAPQ metrics exceeding thresholds of 10, 30, and 50. Across all experimental runs, the use of the modified index consistently yielded a decrease in the number of multi-mapped reads.

k-mer and window sizes	MAPQ	% mapped reads with certain MAPQ		
		Minimap2 with default index	Minimap2 with modified index (VCF from 1000 Genomes Project)	Minimap2 with modified index (VCF from HPRC)
k 15, w 8	0 (multi-map)	2.635	2.634	2.606
	>= 10	92.128	92.154	91.782
	>= 30	90.090	90.144	89.903
	>= 50	87.901	87.982	87.796
k 21, w 11	0 (multi-map)	2.848	2.829	2.800
	>= 10	92.120	92.152	91.773
	>= 30	90.614	90.663	90.389
	>= 50	88.830	88.913	88.708
k 27, w 14	0 (multi-map)	3.228	3.207	3.161
	>= 10	91.671	91.717	91.347
	>= 30	90.328	90.392	90.093
	>= 50	88.597	88.688	88.464

Supplementary Table 3. The evaluation of the acquired VCF files against the reference was performed within "confident regions", utilizing HG002 raw reads and reference data sourced from PrecisionFDA Truth Challenge V2 aligned to the linear reference sequence GRCh38 (GCA_000001405.15).

Tool	Minimap2 with default index			Minimap2 with modified index (VCF from 1000 Genomes Project)			Minimap2 with modified index (VCF from HPRC)			Bowtie 2	BWA-MEM	BWA-MEM2
SNVs in index	–	–	–	6833788			20689457			–	–	–
Indels in index	–	–	–	843004			7445607			–	–	–
k-mer and window	k 15, w 8	k 21, w 11	k 27, w 14	k 15, w 8	k 21, w 11	k 27, w 14	k 15, w 8	k 21, w 11	k 27, w 14	–	–	–
Parameter f	1033, 7368	1000, 5000	607, 3621	1072, 7528	1044, 5195	633, 3791	1141, 7590	1127, 5578	683, 4098	–	–	–
Minimizers in index	673735728	490565423	393318372	698666370	515149313	418775210	743375725	561644550	466504645	–	–	–
Run time	96m 26.489s	45m 52.717s	31m 48.354s	86m 29.813s	46m 59.489s	31m 56.325s	125m 58.7s	56m 29.470s	37m 7.072s	86m 16.386s	31m 35.646s	21m 29.554s
**SNVs**												
TP	3330615	3334220	3334076	3332000	3335444	3336158	3338509	3342362	3343575	3248777	3239702	3339366
FN	34512	30907	31051	33127	29683	28969	26618	22765	21552	116350	125425	25761
FP	21526	20761	17957	21488	20665	17757	18446	17092	14248	12977	24485	36201
Recall	0.98974	0.99082	0.99077	0.99016	0.99118	0.99139	0.99209	0.99324	0.99360	0.96543	0.96273	0.99235
Precision	0.99358	0.99381	0.99464	0.99359	0.99384	0.99471	0.99451	0.99491	0.99576	0.99602	0.99250	0.98928
F1-Score	0.99166	0.99231	0.99270	0.99187	0.99251	0.99304	0.99330	0.99407	0.99468	0.98048	0.97739	0.99081
**Indels**												
TP	520316	520264	520452	520314	520487	520527	520454	520612	520657	514138	499040	520384
FN	5153	5205	5017	5155	4982	4942	5015	4857	4812	11331	26429	5085
FP	4311	4318	3705	4320	4112	3678	4031	3759	3391	3212	10668	5454
Recall	0.99019	0.99001	0.99045	0.99019	0.99052	0.99060	0.99046	0.99076	0.99084	0.97844	0.94970	0.99032
Precision	0.99179	0.99178	0.99293	0.99177	0.99217	0.99299	0.99232	0.99284	0.99353	0.99379	0.97908	0.98963
F1-Score	0.99099	0.99093	0.99169	0.99098	0.99134	0.99179	0.99139	0.99180	0.99219	0.98606	0.96417	0.98998

Supplementary Table 4. The assessment of the generated VCF files against the reference was performed by utilizing HG002 raw reads and reference data sourced from PrecisionFDA Truth Challenge, aligned to the linear reference sequence GRCh19 (hs37d5).

Tool	Minimap2 with default index			Minimap2 with modified index (VCF from 1000 Genomes Project)			Minimap2 with modified index (VCF from HPRC)			Bowtie 2	BWA-MEM	BWA-MEM2
SNVs in index	–	–	–	7609482			20405885			–	–	–
Indels in index	–	–	–	1193661			7355511			–	–	–
k-mer and window	k 15, w 8	k 21, w 11	k 27, w 14	k 15, w 8	k 21, w 11	k 27, w 14	k 15, w 8	k 21, w 11	k 27, w 14	–	–	–
Parameter f	1374, 6868	1000, 5000	802, 4009	1436, 6898	1055, 5223	858, 4232	1544, 7507	1157, 5654	954, 4669	–	–	–
Minimizers in index	665931402	484793717	388675338	691683015	511183729	416000320	734629788	554938084	460920498	–	–	–
Run time	81m 19.394s	44m 30.907s	41m 32.402s	76m 50.408s	46m 52.459s	41m 50.795s	73m 29.620s	45m 37.125s	42m 32.402s	124m 36.9s	36m 35.207s	28m 25.637s
**SNVs**												
TP	3328366	3330532	3330391	3331282	3333360	334145	3334576	3336872	3337920	3352686	3247634	3333551
FN	24320	22154	22295	21404	19326	18541	18110	15814	14766	112964	105052	19135
FP	17079	15809	15921	15897	14377	14289	15602	13790	13708	42316	9749	21324
Recall	0.99275	0.99339	0.99335	0.99361	0.994236	0.9945	0.9946	0.99528	0.99560	0.96631	0.96867	0.99429
Precision	0.99489	0.99528	0.99524	0.99525	0.99571	0.99573	0.99534	0.99588	0.99591	0.98711	0.99701	0.99364
F1-Score	0.99382	0.99433	0.9943	0.99443	0.99497	0.9951	0.99497	0.99558	0.99575	0.97660	0.98263	0.99397
**Indels**												
TP	518288	518267	518306	518376	518381	518465	518431	518413	518493	511668	503488	518270
FN	4102	4123	4084	4014	4009	3925	3959	3977	3897	10722	18902	4120
FP	2948	3008	2957	2843	2896	2840	2760	1783	2704	5198	7251	3226
Recall	0.99215	0.99211	0.99218	0.99232	0.99233	0.99249	0.99242	0.99238	0.99254	0.97948	0.96382	0.99211
Precision	0.99435	0.99423	0.99433	0.99455	0.99445	0.99456	0.99471	0.99475	0.99481	0.98995	0.98581	0.99382
F1-Score	0.99325	0.99317	0.99326	0.99343	0.99339	0.99352	0.99356	0.99357	0.99368	0.98468	0.97469	0.99296

Supplementary Table 5. The assessment of the generated VCF files against the reference was performed by utilizing HG002 reference data from the PrecisionFDA Truth Challenge, aligned to the linear reference sequence hs37d5. Synthetic reads generated by HG002 benchmark VCF file using ART toolset.

Reference	Chromosome 1			Chromosome 6		
Tool	Minimap2 with default index	Minimap2 with modified index (VCF from 1000 Genomes Project)	Minimap2 with modified index (VCF from HPRC)	Minimap2 with default index	Minimap2 with modified index (VCF from 1000 Genomes Project)	Minimap2 with modified index (VCF from HPRC)
**SNVs**						
TP	236673	236915	236945	183613	183886	183926
FN	1215	973	943	1167	894	854
FP	2434	2438	2456	3005	2992	3016
Recall	0.99389	0.99591	0.99604	0.99368	0.99516	0.99538
Precision	0.98982	0.98981	0.98974	0.98390	0.98399	0.98387
F1-Score	0.99235	0.99285	0.99288	0.98877	0.98954	0.98959
**Indels**						
TP	24092	24097	24093	19537	19538	19536
FN	1985	1980	1984	1413	1412	1414
FP	2857	2847	2847	2160	2154	2151
Recall	0.92388	0.92407	0.92392	0.93255	0.93260	0.93251
Precision	0.89391	0.89426	0.89424	0.90040	0.90066	0.90077
F1-Score	0.90865	0.90892	0.90884	0.91620	0.91635	0.91636

Supplementary Table 6. The evaluation of the acquired VCF files against the reference was performed within "confident regions", utilizing HG002 raw reads and reference data sourced from PrecisionFDA Truth Challenge V2 aligned to the linear reference sequence GRCh38 (GCA_000001405.15). Index modified with the addition of population-specific mutations from gnomAD v3.1.2.

Added population	Ashkenazi Jewish	East Asian	Admixed American
SNVs in index	311393	487442	158018
Indels in index	140140	200459	62070
k-mer and window	k27, w14		
Parameter f	609, 3630	609, 3636	607, 3626
Minimizers in index	393913199	394503637	395195323
Run time	36m 7.888s	35m 58.938s	41m 13.455s
**SNVs**			
TP	3334248	3334143	3334087
FN	30879	30984	31040
FP	18235	18596	18244
Recall	0.99082	0.99079	0.99078
Precision	0.99456	0.99445	0.99456
F1-Score	0.99269	0.99262	0.99266
**Indels**			
TP	520451	520441	520455
FN	5018	5028	5014
FP	3729	3765	3715
Recall	0.99045	0.99043	0.99046
Precision	0.99289	0.99282	0.99291
F1-Score	0.99167	0.99163	0.99169

Supplementary Figure 1-2. The genetic variants found using the modified index. There are 1 SNV (chr1: 58,273,219) and 1 deletion (chr1: 58,273,237).

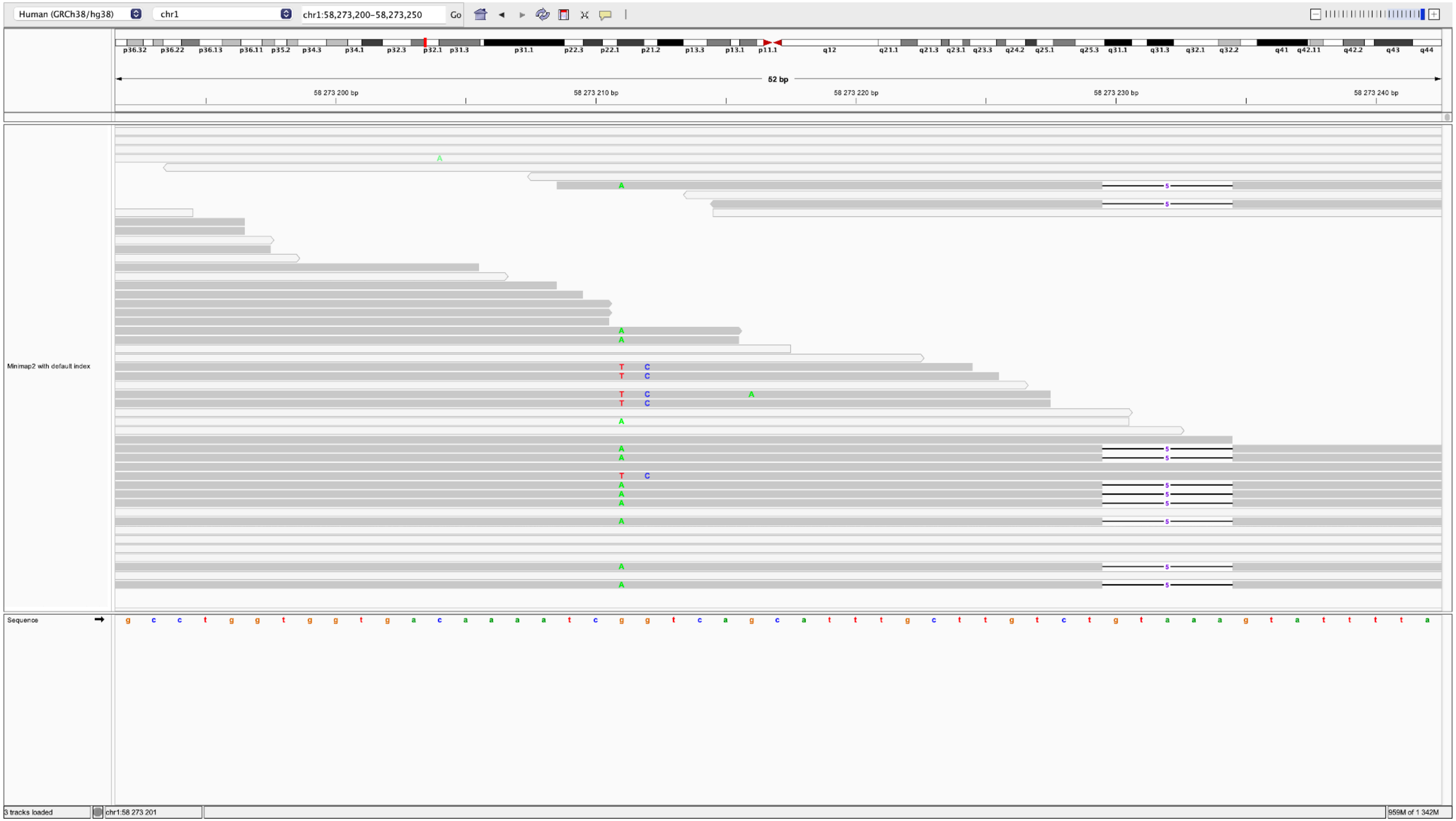

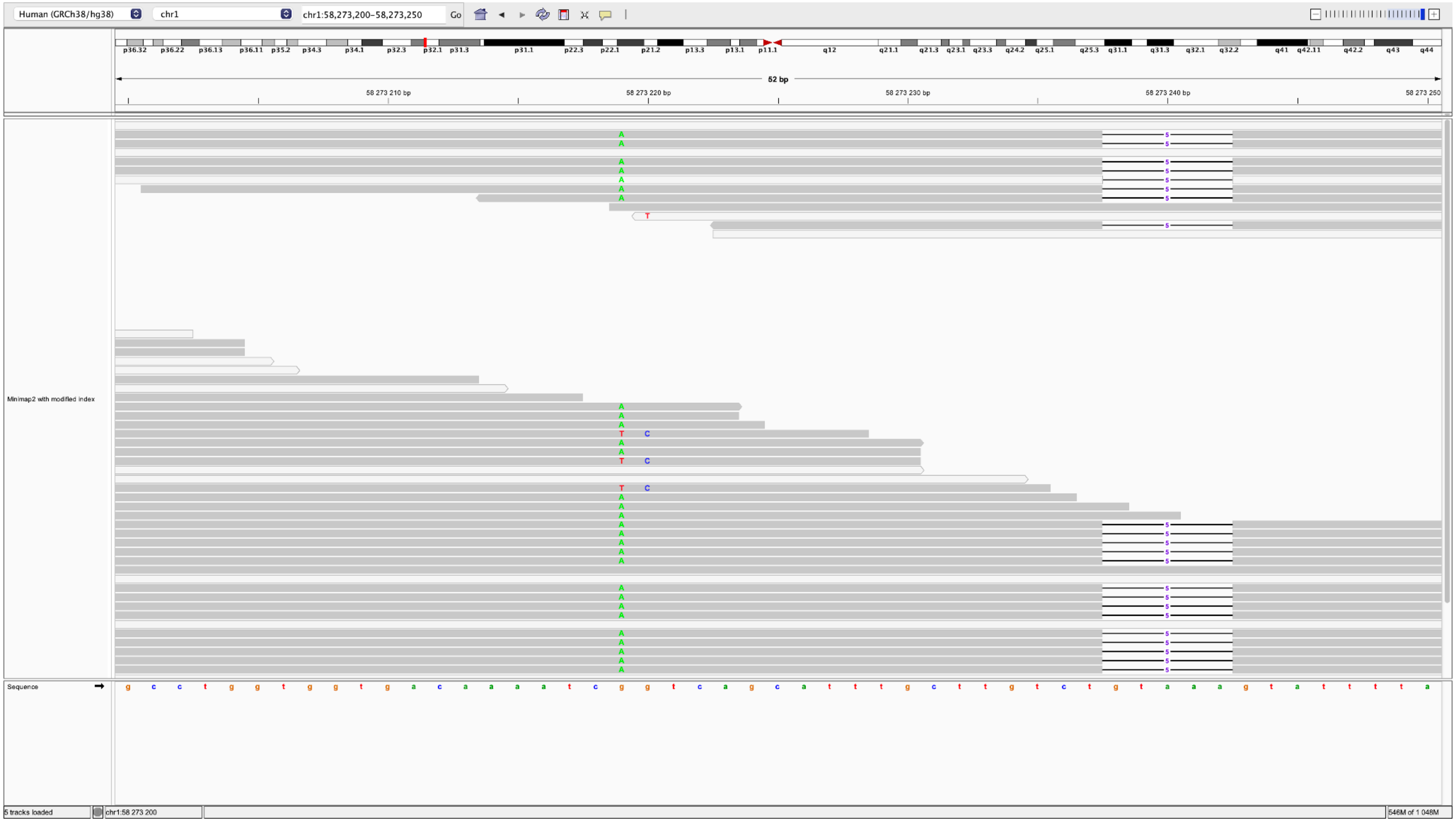
Human (GRCh38/hg38)
chr1
chr1:58,273,200-58,273,250
Go
52 bp
58 273 210 bp
58 273 220 bp
58 273 230 bp
58 273 240 bp
58 273 250
Minimap2 with modified index
Sequence
5 tracks loaded
chr1:58 273 200
546M of 1 048M

Supplementary methods

1.Data

1.1 Sequencing data in FASTQ format

(HG002 35x)
https://precision.fda.gov/home/files/file-FpZG9Jj0xbJy0QXjB7yb6fpX-1
https://precision.fda.gov/home/files/file-FpZG9Jj0xbJfGbf01qBgKyg1-1

(HG002 50x)
https://precision.fda.gov/home/files/file-BvP0b3Q0Z8pJfQxYxbk8P2p0
https://precision.fda.gov/home/files/file-BvP0f600xKVyPQZj08ZjXPx4

1.2 Benchmark variant calls in VCF file

(HG002 GRCh38 v4.2.1)
https://ftp-trace.ncbi.nlm.nih.gov/giab/ftp/release/AshkenazimTrio/HG002_NA24385_son/NISTv4.2.1/GRCh38/HG002_GRCh38_1_22_v4.2.1_benchmark.vcf.gz
https://ftp-trace.ncbi.nlm.nih.gov/giab/ftp/release/AshkenazimTrio/HG002_NA24385_son/NISTv4.2.1/GRCh38/HG002_GRCh38_1_22_v4.2.1_benchmark.vcf.gz.tbi

(HG002 GRCh37 v4.2.1)
https://ftp-trace.ncbi.nlm.nih.gov/giab/ftp/release/AshkenazimTrio/HG002_NA24385_son/NISTv4.2.1/GRCh37/HG002_GRCh37_1_22_v4.2.1_benchmark.vcf.gz
https://ftp-trace.ncbi.nlm.nih.gov/giab/ftp/release/AshkenazimTrio/HG002_NA24385_son/NISTv4.2.1/GRCh37/HG002_GRCh37_1_22_v4.2.1_benchmark.vcf.gz.tbi

1.3 Confident regions in BED file

(HG002 GRCh38 v4.2.1)
https://ftp-trace.ncbi.nlm.nih.gov/giab/ftp/release/AshkenazimTrio/HG002_NA24385_son/NISTv4.2.1/GRCh38/HG002_GRCh38_1_22_v4.2.1_benchmark_noinconsistent.bed

(HG002 GRCh37 v4.2.1)
https://ftp-trace.ncbi.nlm.nih.gov/giab/ftp/release/AshkenazimTrio/HG002_NA24385_son/NISTv4.2.1/GRCh37/HG002_GRCh37_1_22_v4.2.1_benchmark_noinconsistent.bed

1.4 1000 Genomes Project phase 3 VCF files

(GRCh38)
Downloaded from:
ftp://hgdownload.soe.ucsc.edu/gbdb/hg38/1000Genomes/

(GRCh37)
Downloaded from:
http://hgdownload.cse.ucsc.edu/gbdb/hg19/1000Genomes/phase3/

1.5 HPRC v1.1 VCF file

Downloaded from:
https://s3-us-west-2.amazonaws.com/human-pangenomics/pangenomes/freeze/freeze1/minigraph-cactus/hprc-v1.1-mc-grch38/hprc-v1.1-mc-grch38.vcfbub.a100k.wave.vcf.gz
https://s3-us-west-2.amazonaws.com/human-pangenomics/pangenomes/freeze/freeze1/minigraph-cactus/hprc-v1.1-mc-grch38/hprc-v1.1-mc-grch38.vcfbub.a100k.wave.vcf.gz.tbi

1.6 gnomAD v3.1.2 VCF files (GRCh38)

Downloaded from:
https://storage.googleapis.com/gcp-public-data--gnomad/release/3.1.2/vcf/genomes/

1.7 Additional files for Base Quality Score Recalibrator

(GRCh38)
ftp://gsapubftp-anonymous@ftp.broadinstitute.org/bundle/hg38/dbsnp_138.hg38.vcf.gz
ftp://gsapubftp-anonymous@ftp.broadinstitute.org/bundle/hg38/dbsnp_138.hg38.vcf.gz.tbi
ftp://gsapubftp-anonymous@ftp.broadinstitute.org/bundle/hg38/Mills_and_1000G_gold_standard.indels.hg38.vcf.gz
ftp://gsapubftp-anonymous@ftp.broadinstitute.org/bundle/hg38/Mills_and_1000G_gold_standard.indels.hg38.vcf.gz.tbi

(GRCh37)
ftp://gsapubftp-anonymous@ftp.broadinstitute.org/bundle/b37/dbsnp_138.b37.vcf.gz
ftp://gsapubftp-anonymous@ftp.broadinstitute.org/bundle/b37/Mills_and_1000G_gold_standard.indels.b37.vcf.gz

2. Commands

2.1 Command for trimming using cutadapt v1.18

```
cutadapt -q 0,0 --minimum-length 60 -a AGATCGGAAGAGCACACGTCTGAACTCCAGTCA -A AGATCGGAAGAGCGTCGTGTAGGGAAAGAGTGT
--pair-filter=any  -o $output_fastq_file1 -p $output_fastq_file2 $input_fastq_file1 $input_fastq_file2
```

2.2 Command for minimap2 index creation using minimap2_index_modifier (minimap2 v2.24)

```
minimap2 -d $output_index -k $k -w $w --vcf-file-with-variants $input_vcf [--parse-haplotype] $ref_fasta
```

2.3 Commands for read alignment with minimap2 (minimap2 v2.24)

```
minimap2 -a -Y -f $n,$m -x sr -R '@RG\tID:HG002\tSM:HG002\tPL:ILLUMINA’ $modified_index $trimmed_fastq1 $trimmed_fastq2  > $output_intermediate_bam
```

2.4 Commands for BWA-MEM index creation (BWA-MEM v0.7.17)

```
bwa index $ref_fasta
```

2.5 Commands for read alignment with BWA-MEM (BWA-MEM v0.7.17)

```
bwa mem -K 100000000 -p -v 3 -Y -R '@RG\tID:HG002\tSM:HG002\tPL:Illumina' \
$ref_fasta  $trimmed_fastq1 $trimmed_fastq2 > $output_intermediate_bam
```

2.6 Commands for BWA-MEM 2 index creation (BWA-MEM 2 v2.2.1)

```
bwa-mem2 index $ref_fasta
```

2.7 Commands for read alignment with BWA-MEM 2 (BWA-MEM 2 v2.2.1)

```
bwa-mem2 -K 100000000 -v 3 -Y -R '@RG\tID:HG002\tSM:HG002\tPL:Illumina' \
$ref_fasta  $trimmed_fastq1 $trimmed_fastq2 > $output_intermediate_bam
```

2.8 Commands for Bowtie2 index creation (Bowtie 2 v2.5.2)

```
bowtie2-build $ref_fasta $ref_fasta_name
```

2.9 Commands for read alignment with Bowtie 2 (Bowtie 2 v2.5.2)

```
bowtie2 -x $index_basename --rg-id @RG\tID:HG002 --rg SM:HG002 --rg PL:BGI \
-1 $trimmed_fastq1 -2 $trimmed_fastq2 > $output_intermediate_bam
```

2.10 Command for BAM duplicate removal, sorting, and indexing (samtools v1.18)

```
samtools fixmate -m -O SAM $output_intermediate_bam - |  samtools sort - | samtools markdup -d 100  - $output_bam
samtools index $output_bam $output_bam_index
```

2.11 Command for Base Quality Score Recalibration (GATK v4.2.6.1, samtools v1.18)

```
gatk BaseRecalibrator \
   -R $ref_fasta \
   -I $input_bam \
   --read-index $input_bam_index \
   --use-original-qualities \
   -O $report_file \
   --known-sites $dbsnp \
   --known-sites $mills_and_1000g
gatk ApplyBQSR \
   --add-output-sam-program-record \
   -R $ref_fasta \
   -I $input_bam \
   --read-index $input_bam_index \
   --use-original-qualities \
   -O $output_recalibrated_bam \
   -bqsr $report_file \
   --static-quantized-quals 10 \
   --static-quantized-quals 20 \
   --static-quantized-quals 30

samtools index $output_recalibrated_bam $output_bam_recalibrated_index
```

2.12 Command for variant calling (GATK v4.2.6.1)

```
gatk HaplotypeCaller \
    -R $ref_fasta \
    -I $input_recalibrated_bam \
    --read-index $input_recalibrated_bam_index \
    -O $output_vcf \
    -contamination 0 \
    --native-pair-hmm-threads 1
```

2.13 Command for VCF files comparison (hap.py v0.3.15)

```
hap.py $benchmark_vcf $ result_vcf -f $confident_regions_bed -r $ref_fasta \
-V --engine=vcfeval --no-leftshift --no-decompose --gender=none
```

2.14 Commands for synthetic read generation (bcftools v1.19, ART v2.5.8)

(example for chromosome 1, same commands for chromosome 6)

```
bcftools consensus -H R -f $input_fasta_chr1 $input_vcf_chr1 > $out_fasta1
bcftools consensus -H A -f $input_fasta_chr1 $input_vcf_chr1 > $out_fasta2
art_illumina -ss HS25 -sam -i  $out_fasta1 -p -l 150 -f 19 -m 200 -s 10 -qs 30 -qs2 30 -o ${prefix1}
art_illumina -ss HS25 -sam -i  $out_fasta2 -p -l 150 -f 19 -m 200 -s 10 -qs 30 -qs2 30 -o ${prefix2}
cat ${prefix1}1.fq ${prefix2}1.fq > $output_fq1
cat ${prefix1}2.fq ${prefix2}2.fq > $output_fq2
```

3 Source code repository

minimap2_index_modifier repository is a fork of the original minimap2 repository (https://github.com/lh3/minimap2).
All functions from linked_vcf_list.c file are our contributions as well as read_vcf, mm_idx_manipulate_phased, add_indel, add_variants functions from sketch.c.

4 Algorithms

Algorithm 4.1: Minimizer search for the reference genetic sequences

Algorithm 3.1 Minimizer search for the reference genetic sequences

Input: w – window size, k – kmer length, s – sequence ($|s| \geq w + k - 1$)
Output: (w, k) – minimizers and their positions

```
function GetMinimizers(s, k, w)
    M ← ∅
    for i = 1 to |s| − w − k − 1 do                ▷ Find minimum value
        m ← ∞
        for j = 0 to w − 1 do
            u ← s_i + j_k
            v ← s̄_i + j_k                          ▷ s̄ – reversed strand
            if u ≠ v then
                m = min(m, min(u, v))
            end if
        end for
        for j = 0 to w − 1 do                      ▷ Collect minimizers
            u ← s_i + j_k
            v ← s̄_i + j_k
            if u < v and u = m then
                M ← M ∪ (m, i + j, 0)
            end if
            if u > v and v = m then
                M ← M ∪ (m, i + j, 1)
            end if
        end for
    end for
    return M
end function
```

Algorithm 4.2: Additional minimizer search for SNV

Algorithm 3.2 Additional minimizer search for SNV

Input: w – window size, k – kmer length, s – sequence ($|s| \geq 2 \cdot (w+k) - 1$), (var, pos) – variant and its position

Output: (w, k) – minimizers and their positions

function GetAdditionalMinimizers(s, k, w, var, pos)
    $subs \leftarrow s[pos - k - w + 1] \ldots s[pos + k + w - 1]$
    $subs[k + w - 1] \leftarrow var$ ▷ Modify sequence with SNV
    $M \leftarrow \varnothing$
    for $i = 0$ to $2 \cdot (w + k - 1)$ **do** ▷ Find minimum value
        $m \leftarrow \infty$
        for $j = 0$ to $w - 1$ **do**
            $u \leftarrow subs_i + j_k$
            $v \leftarrow \overline{subs}_i + j_k$ ▷ $\overline{subs}$ – reversed strand
            if $u \neq v$ **then**
                $m = min(m, min(u, v))$
            end if
        end for
        for $j = 0$ to $w - 1$ **do** ▷ Collect minimizers
            $u \leftarrow subs_i + j_k$
            $v \leftarrow \overline{subs}_i + j_k$
            if $u < v$ and $u = m$ **then**
                $M \leftarrow M \cup (m, i + j, 0)$
            end if
            if $u > v$ and $v = m$ **then**
                $M \leftarrow M \cup (m, i + j, 1)$
            end if
        end for
    end for
    return M
end function

Algorithm 4.3: Searching valid combinations of SNVs in a window of length k

Algorithm 3.3 Searching valid combinations of SNVs in a window of length k

Input: *genotypes* – list of SNVs' genotypes, $begin_{ptr}$, end_{ptr} – pointers to SNVs in current window

Output: *Combs* – array of SNVs combinations in a window of length k

function GETCOMBS(s, k, w)
    $Combs \leftarrow \varnothing$
    $Size \leftarrow size(genotypes)$
    for $i = 0$ to $Size - 1$ **do**
        $w_{ptr} \leftarrow begin_{ptr}$
        $CombsWithPos \leftarrow \varnothing$
        while $w_{ptr} \neq end_{ptr}$ **do**
            if $w_{ptr} \rightarrow genotypes[i] = 0$ **then**
                $CombsWithPos \cup (w_{ptr} \rightarrow allele_{ref}, w_{ptr} \rightarrow pos)$
            end if
            if $w_{ptr} \rightarrow genotypes[i] = 1$ **then**
                $CombsWithPos \cup (w_{ptr} \rightarrow allele_{alt}, w_{ptr} \rightarrow pos)$
            end if
            $w_{ptr} \leftarrow w_{ptr} \rightarrow next$
        end while
        $Combs \cup CombsWithPos$
    end for
    return *Combs*
end function
